# Supplementary material for: Common AAV gene therapy vectors show indiscriminate transduction of living human brain cell types
Source: bioRxiv. 2024 Nov 15:2024.11.14.623624. Preprint. [Version 1] doi: 10.1101/2024.11.14.623624 (PMC11601464; doi:10.1101/2024.11.14.623624)
Supplement: Supplement 1 [file NIHPP2024.11.14.623624v1-supplement-1.pdf]

bioRxiv preprint doi: https://doi.org/10.1101/2024.11.14.623624; this version posted November 15, 2024. The copyright holder for this preprint (which was not certified by peer review) is the author/funder, who has granted bioRxiv a license to display the preprint in perpetuity. It is made available under aCC-BY 4.0 International license.

Supplemental Figure 1

A

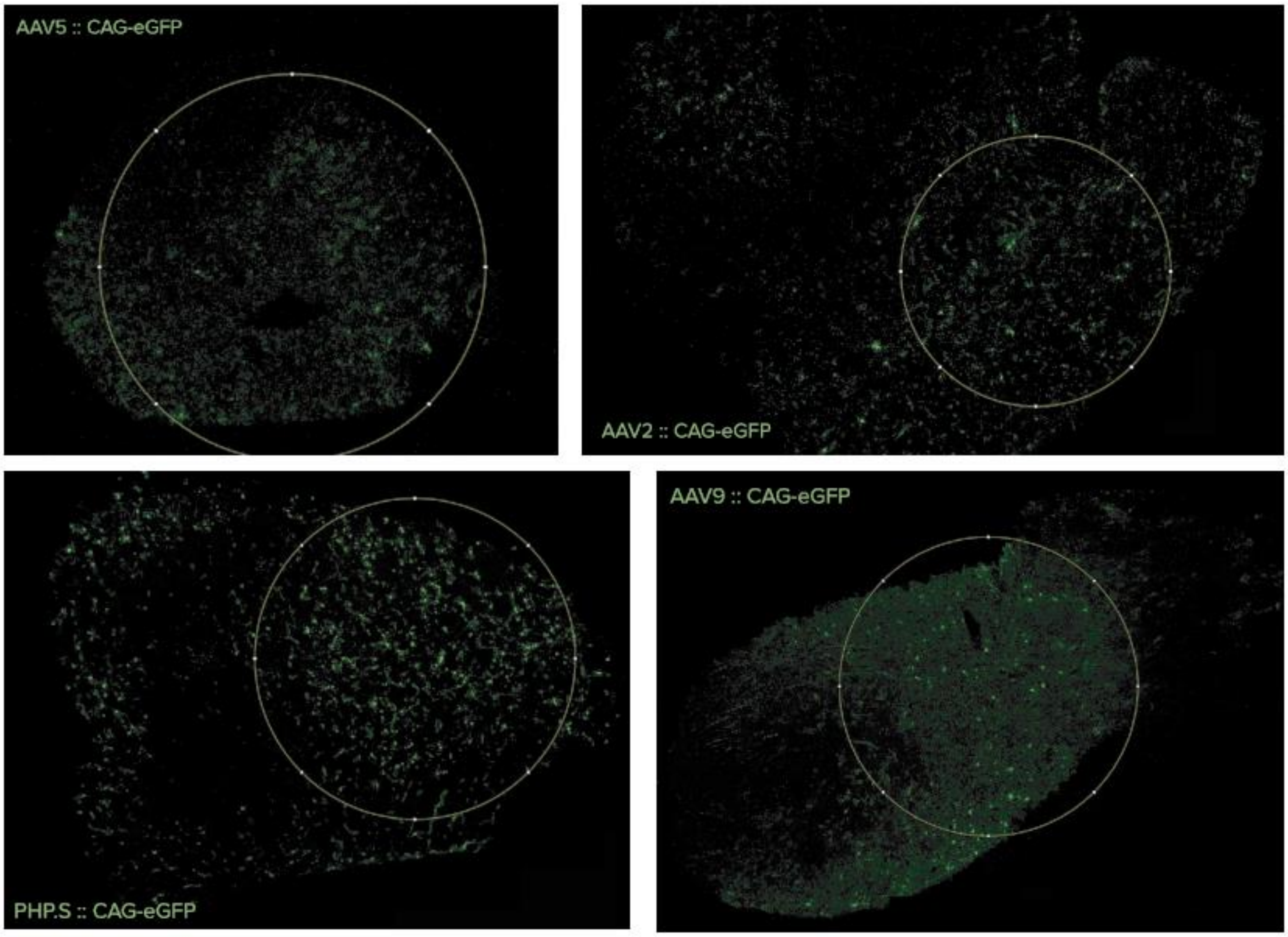

B

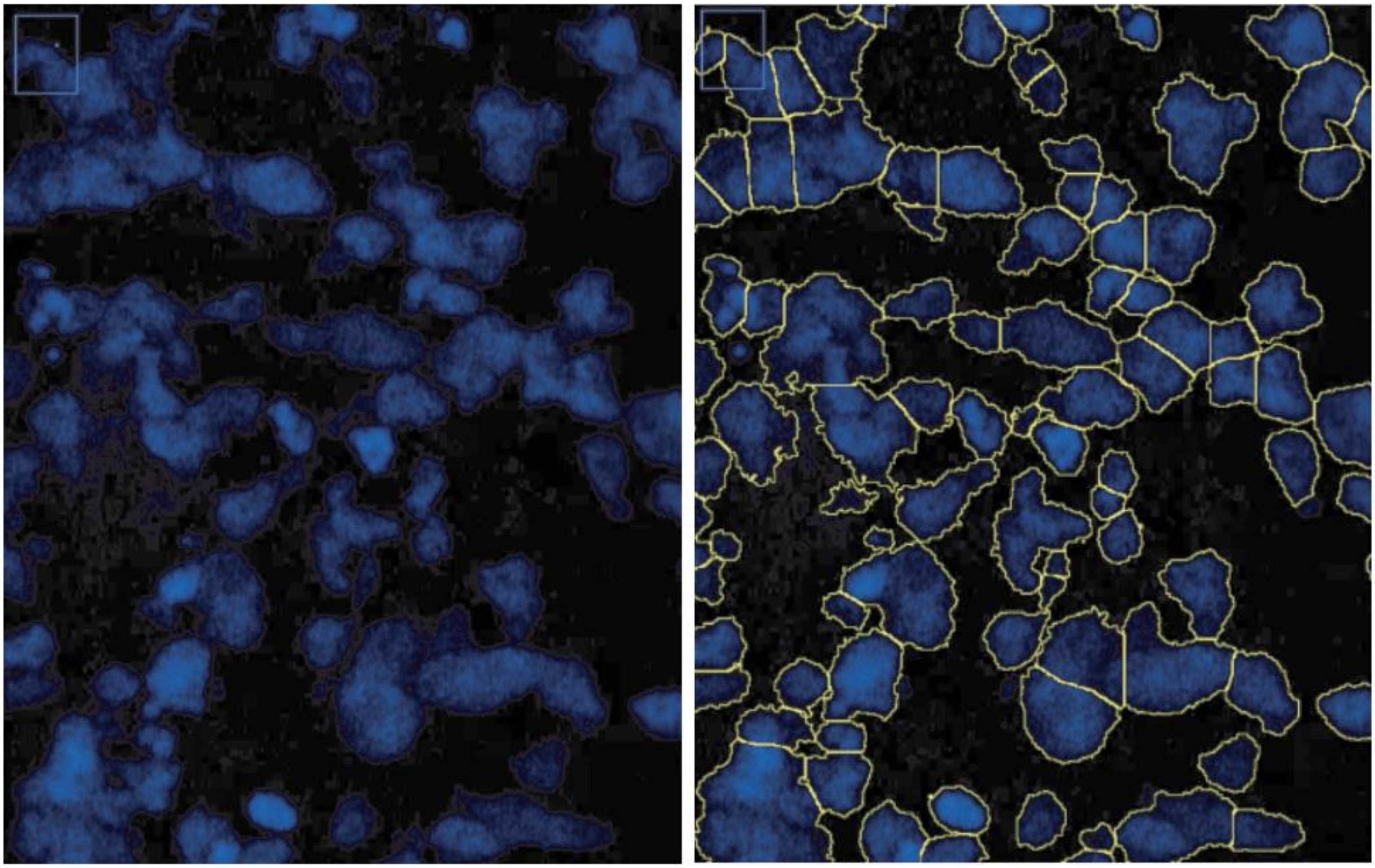

bioRxiv preprint doi: https://doi.org/10.1101/2024.11.14.623624; this version posted November 15, 2024. The copyright holder for this preprint (which was not certified by peer review) is the author/funder, who has granted bioRxiv a license to display the preprint in perpetuity. It is made available under aCC-BY 4.0 International license.

Supplemental Figure 1 | Selection of regions of interest (ROI) for image analysis. a, Example screenshots of the 2mm circular region used to restrict analysis to the highest 2mm diameter circlular region of each slice. **b,** Representative images showing the custom automated DAPI-centric ROI identification. The average intensity within each ROI is separately calculated for each of the four channels.

bioRxiv preprint doi: https://doi.org/10.1101/2024.11.14.623624; this version posted November 15, 2024. The copyright holder for this preprint (which was not certified by peer review) is the author/funder, who has granted bioRxiv a license to display the preprint in perpetuity. It is made available under aCC-BY 4.0 International license.

Supplemental Figure 2

a.

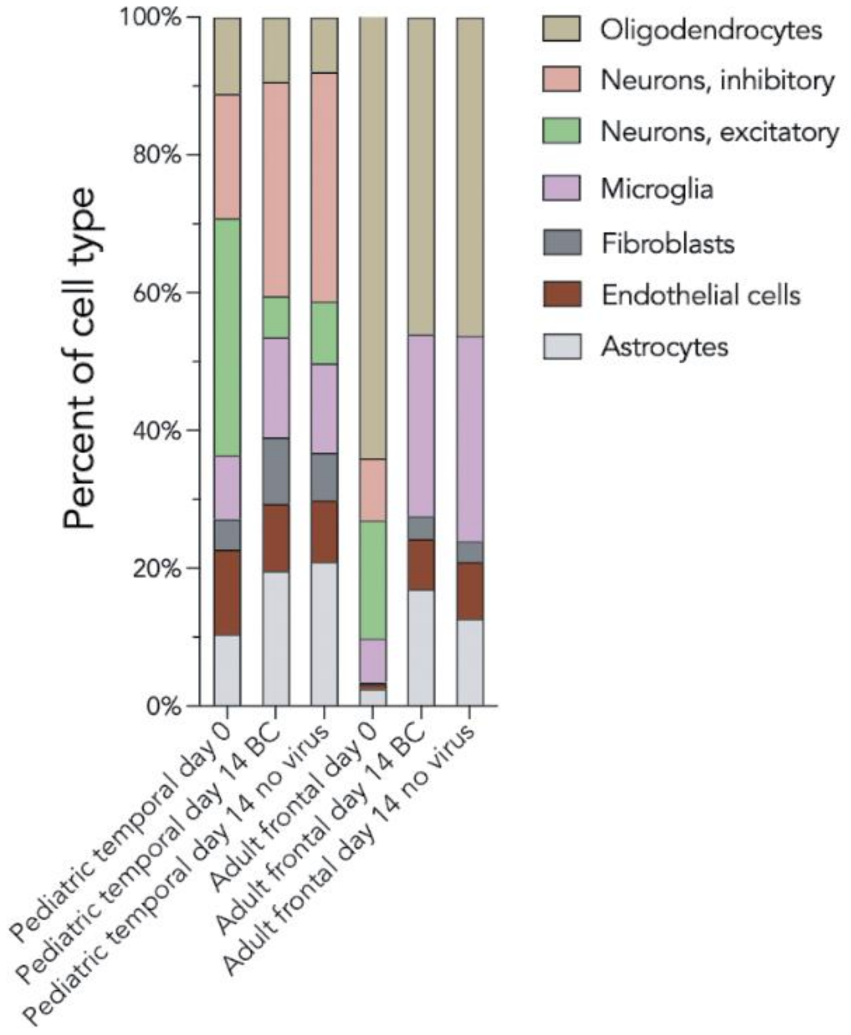

b.

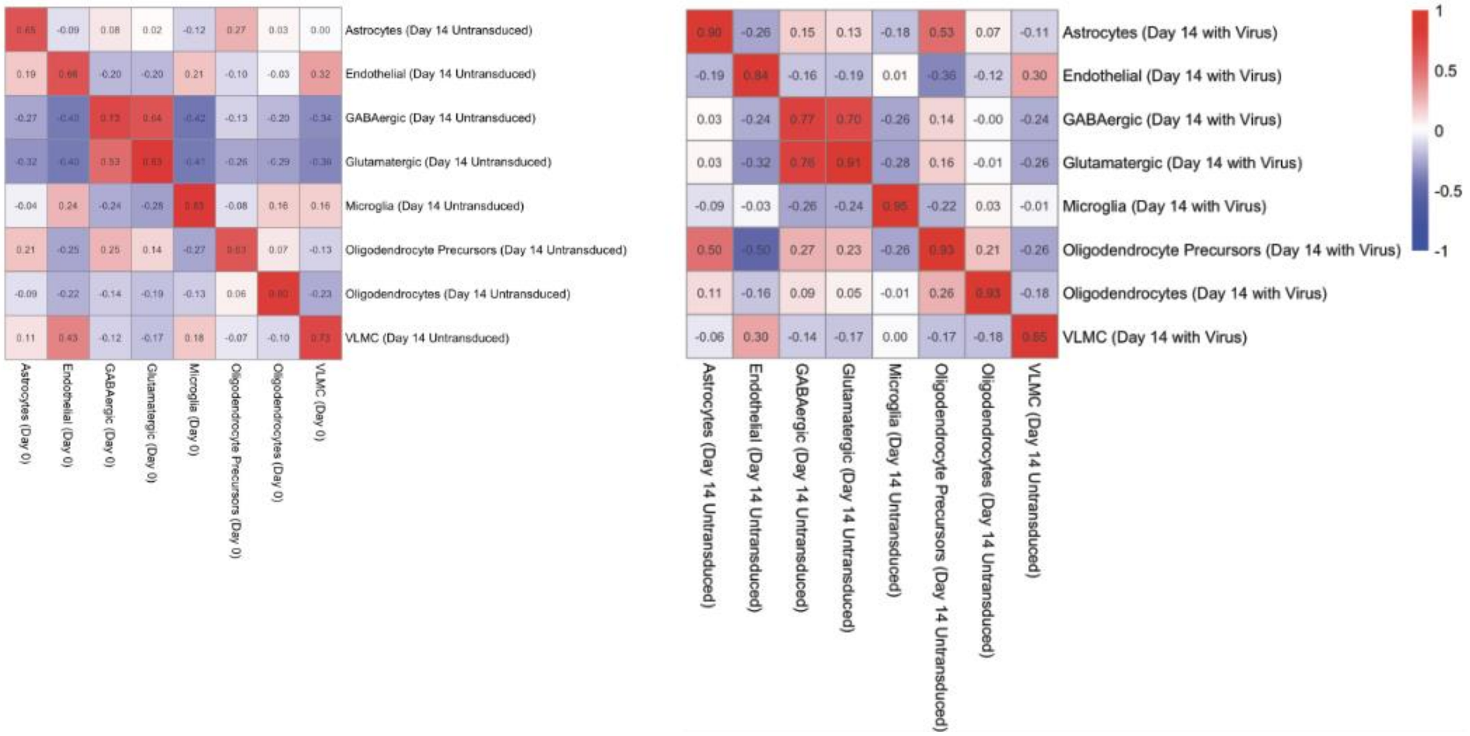

bioRxiv preprint doi: https://doi.org/10.1101/2024.11.14.623624; this version posted November 15, 2024. The copyright holder for this preprint (which was not certified by peer review) is the author/funder, who has granted bioRxiv a license to display the preprint in perpetuity. It is made available under aCC-BY 4.0 International license.

Supplemental Figure 2 | Cell type composition changes across fourteen days. a, Proportions of cell types present in sequenced tissues at each time point, with and without virus. **b**, Correlation matrices (correlation calculated across all differentially expressed genes identified for that sample's clusters) for day 0 versus day 14 without virus, and for day 14 with virus versus day 14 without virus. Pearson correlation coefficients are printed within each cell.

bioRxiv preprint doi: https://doi.org/10.1101/2024.11.14.623624; this version posted November 15, 2024. The copyright holder for this preprint (which was not certified by peer review) is the author/funder, who has granted bioRxiv a license to display the preprint in perpetuity. It is made available under aCC-BY 4.0 International license.

Supplemental Figure 3

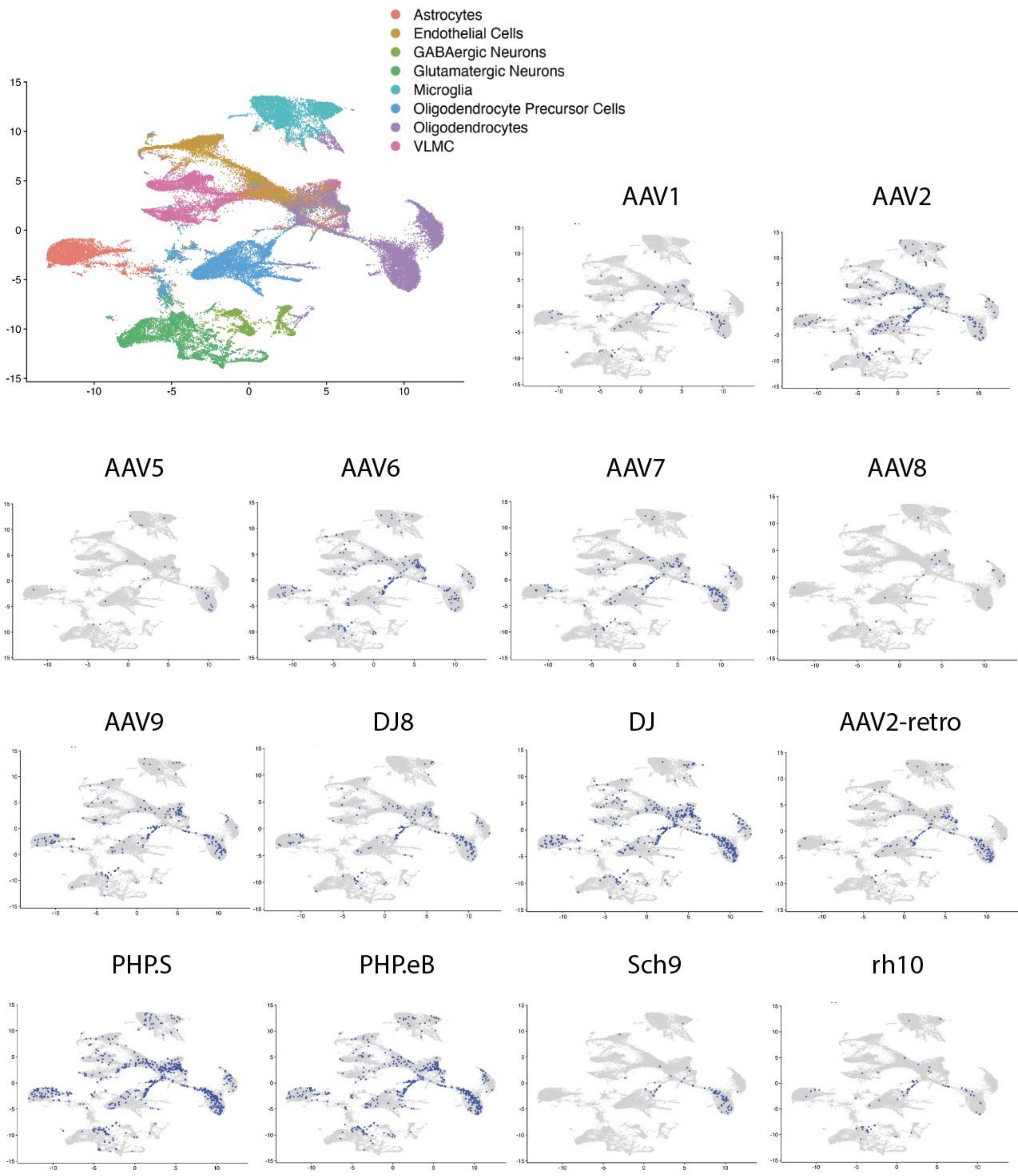

bioRxiv preprint doi: https://doi.org/10.1101/2024.11.14.623624; this version posted November 15, 2024. The copyright holder for this preprint (which was not certified by peer review) is the author/funder, who has granted bioRxiv a license to display the preprint in perpetuity. It is made available under aCC-BY 4.0 International license.

Supplemental Figure 3 | All AAV capsid variants show broad transduction across cell types.

UMAPs showing the transduction profiles of each AAV capsid variant across cell types (purple dot = cells with at least one viral transcript detected). All show transduction of numerous cell types.

Supplemental Table 1 | AAV capsid, cargo, and barcode sequences used. A table with the fourteen Cap sequences used in this study, the ITR-CAG-eGFP-WPRE-hGH-ITR (modified from Addgene 37825, gift from Ed Boyden), and barcodes, corresponding to their respective capsid variants, which were placed in between the WPRE and hGH sequences at the NotI restriction site.

Capsid	Sequence
AAV1	atggctgccgatggttatcttccagattggctcgaggacaacctctctgagggcattcgcgagtggtgggacttgaaacctggagccccgaagcccaaagccaacc<br>agcaaaagcaggacgacggccggggtctggtgcttcctggctacaagtacctcggacccttcaacggactcgacaagggggagcccgtcaacgcggcggacgca<br>gcggccctcgagcacgacaaggcctacgaccagcagctcaaagcgggtgacaatccgtacctgcggtataaccacgccgacgccgagtttcaggagcgtctgca<br>agaagatacgtcttttgggggcaacctcgggcgagcagtcttccaggccaagaagcgggttctcgaacctctcggtctggttgaggaaggcgctaagacggctcct<br>ggaaagaaacgtccggtagagcagtcgccacaagagccagactcctcctcgggcatcggcaagacaggccagcagcccgctaaaaagagactcaattttggtca<br>gactggcgactcagagtcagtccccgatccacaacctctcggagaacctccagcaacccccgctgctgtgggacctactacaatggcttcaggcggtggcgcacca<br>atggcagacaataacgaaggcgccgacggagtgggtaatgcctcaggaaattggcattgcgattccacatggctgggcgacagagtcatcaccaccagcacccg<br>cacctgggccttgcccacctacaataaccacctctacaagcaaatctccagtgcttcaacgggggccagcaacgacaaccactacttcggctacagcaccccctgg<br>gggtattttgatttcaacagattccactgccacttttcaccacgtgactggcagcgactcatcaacaacaattggggattccggcccaagagactcaacttcaaactc<br>ttcaacatccaagtcaaggaggtcacgacgaatgatggcgtcacaaccatcgctaataaccttaccagcacggttcaagtcttctcggactcggagtaccagcttc<br>cgtacgtcctcggctctgcgcaccagggctgcctccctccgttcccggcggacgtgttcatgattccgcaatacggctacctgacgctcaacaatggcagccaagcc<br>gtgggacgttcatccttttactgcctggaatatttcccttctcagatgctgagaacgggcaacaactttaccttcagctacacctttgaggaagtgcctttccacagca<br>gctacgcgcacagccagagcctggaccggctgatgaatcctctcatcgaccaatacctgtattacctgaacagaactcaaaatcagtccggaagtgcccaaaaca<br>aggacttgctgtttagccgtgggtctccagctggcatgtctgttcagcccaaaaactggctacctggaccctgttatcggcagcagcgcgtttctaaaacaaaaaca<br>gacaacaacaacagcaattttacctggactggtgcttcaaaatataacctcaatgggcgtgaatccatcatcaaccctggcactgctatggcctcacacaaagacg<br>acgaagacaagttctttcccatgagcggtgtcatgatttttggaaaagagagcgccggagcttcaaacactgcattggacaatgtcatgattacagacgaagagg<br>aaattaaagccactaaccctgtggccaccgaaagatttgggaccgtggcagtcaatttccagagcagcagcacagaccctgcgaccggagatgtgcatgctatgg<br>gagcattacctggcatggtgtggcaagatagagacgtgtacctgcagggtcccatttgggccaaaattcctcacacagatggacactttcacccgtctcctcttatg<br>ggcggctttggactcaagaacccgcctcctcagatcctcatcaaaaacacgcctgttcctgcgaatcctccggcggagttttcagctacaaagtttgcttcattcatc<br>acccaatactccacaggacaagtgagtgtggaaattgaatgggagctgcagaaagaaaacagcaagcgctggaatcccgaagtgcagtacacatccaattatgc<br>aaaatctgccaacgttgattttactgtggacaacaatggactttatactgagcctcgccccattggcacccgttaccttacccgtcccctgtaa

AAV2	atggctgccgatggttatcttccagattggctcgaggacactctctctgaaggaataagacagtggtggaagctcaaacctggcccaccaccaccaaagcccgcag<br>agcggcataaggacgacagcaggggtcttgtgcttcctgggtacaagtacctcggacccttcaacggactcgacaagggagagccggtcaacgaggcagacgcc<br>gcggccctcgagcacgacaaagcctacgaccggcagctcgacagcggagacaacccgtacctcaagtacaaccacgccgacgcggagtttcaggagcgccttaa<br>agaagatacgtcttttgggggcaacctcggacgagcagtcttccaggcgaaaaagagggttcttgaacctctgggcctggttgaggaacctgttaagacggctccg<br>ggaaaaaagaggccggtagagcactctcctgtggagccagactcctcctcgggaaccggaaaggcgggccagcagcctgcaagaaaaagattgaattttggtca<br>gactggagacgcagactcagtacctgacccccagcctctcggacagccaccagcagccccctctggtctgggaactaatacgatggctacaggcagtggcgcacc<br>aatggcagacaataacgagggcgccgacggagtgggtaattcctcgggaaattggcattgcgattccacatggatgggcgacagagtcatcaccaccagcaccc<br>gaacctgggccctgcccacctacaacaaccacctctacaaacaaatttccagccaatcaggagcctcgaacgacaatcactactttggctacagcaccccttgggg<br>gtattttgacttcaacagattccactgccacttttcaccacgtgactggcaaagactcatcaacaacaactggggattccgacccaagagactcaacttcaagctctt<br>taacattcaagtcaaagaggtcacgcagaatgacggtacgacgacgattgccaataaccttaccagcacggttcaggtgtttactgactcggagtaccagctcccg<br>tacgtcctcggctcggcgcatcaaggatgcctcccgccgttcccagcagacgtcttcatggtgccacagtatggatacctcaccctgaacaacgggagtcaggcag<br>taggacgctcttcattttactgcctggagtactttccttctcagatgctgcgtaccggaaacaactttaccttcagctacacttttgaggacgttcctttccacagcagc<br>tacgctcacagccagagtctggaccgtctcatgaatcctctcatcgaccagtacctgtattacttgagcagaacaaacactccaagtggaaccaccacgcagtcaa<br>ggcttcagttttctcaggccggagcgagtgacattcgggaccagtctaggaactggcttcctggaccctgttaccgccagcagcgagtatcaaagacatctgcggat<br>aacaacaacagtgaatactcgtggactggagctaccaagtaccacctcaatggcagagactctctggtgaatccgggcccggccatggcaagccacaaggacga<br>tgaagaaaagtttttcctcagagcggggttctcatctttgggaagcaaggctcagagaaaacaaatgtggacattgaaaaggtcatgattacagacgaagagga<br>aatcaggacaaccaatcccgtggctacggagcagtatggttctgtatctaccaacctccagagaggcaacagacaagcagctaccgcagatgtcaacacacaag<br>gcgttcttccaggcatggtctggcaggacagagatgtgtaccttcaggggcccatctgggcaaagattccacacacggacggacattttcacccctctcccctcatg<br>ggtggattcggacttaaacaccctcctccacagattctcatcaagaacaccccggtacctgcgaatccttcgaccaccttcagtgcggcaaagtttgcttccttcatc<br>acacagtactccacgggacaggtcagcgtggagatcgagtgggagctgcagaaggaaaacagcaaacgctggaatcccgaaattcagtacacttccaactaca<br>acaagtctgttaatgtggactttactgtggacactaatggcgtgtattcagagcctcgccccattggcaccagatacctgactcgtaatctgtaa
AAV5	ttaaaggggtcgggtaaggtatcgggttccgataggtctggtggttctgtattccccggtgctgtccggggcaaagtccacaaactgggggtcgttgtagttgtttgt<br>gtactggatctctgggttccacctcttggagttttccttcttgagctcccactccatctccacggtgacctgcccggtgctgtactgggtgatgaagctgctgacgggc<br>acgtccgagaagctggtgatatttccgggcacaggcgtgttcttgatgagcatcatgggcggtgggtgtttgagtccgaatccgcccatggccggagaggggtgaa<br>agtgcgcccccgtctctgggatcttggcccagatgggtccttggaggtacacgtccctctccatccacacgctgccgggcacgatttcctggaggttgtacgtgccgg<br>tcgcgggggcagtggtggagctctggttgttggtggccatctgcccgccgacgttgtacgccacgcggttcaccggctgcgtctcgctctcgctggtgatgagcatgt<br>tgccctcgaggtacgtggcggtggtgcccgggttcgccggctggctgttgaagatcatagtgttctccagggcataggtgttgctgccctggaggttgttggtcatgc<br>cgttcggctgcgggggcacctggtaactcgcgccctcgagctccatcctattggtcgtggcgaaggcgctgacactggcgcggttgaccccggagcccaggttcca<br>gccctgggttcggcccatgggcccgggaaccagtttttgtaggtgttggcgtatctcccggccaggttcttgttgaactggactccgccagtgttatttgtgctcacg<br>aagcggtacaagtactggtccaccagcgggttggccagcttgaacaggttctgactgggagcgaagctggagtggaagggcacctcctcaaagttgtaggtaaac<br>tcaaagttgttgcccgttctcagcatcttgctgggaaagtactctaggcagaagaagctgctcctctcggtgggattttctgtgttgtcgcggttcagcgtcgcgtaac

	cgtactgcggcagcgtaaagacctgcggagggaaggccggcaggcatccctcggtcccgttgccgacgacgtagggcagctggtagtcgtcgtccgtaaacactt<br>ggacggtggaggtgaggttgttggcgatggtggtggtggagtcctgcaccgtgacctctttgacttgaatgttgaagattttgactctgagggaccggggtctgaag<br>ccccagtagttgttgatgagtctttgccagtctcgggggctccagtggctgtggaagcggttaaagtcaaagtacccccaggggggtgctgtatccaaagtaggcgtt<br>ggcgttgcttccgtcgacggagccgcttttgatctctcggtactggtggttgttgtagctgggcagcacccaggttcgggtggacttggtgacgactctgtcccccatc<br>cacgtggaatcgcaatgccaatctcccgaggcattgcccactccatcggcaccttggttattgtcgcccaatgggccgccacctcccgcagacattgtatcagctccc<br>aaacttgaggctggttgggctgggatttgcagctgctgggatccgctgggtccagcttcggcgtctgacgaggtggaaggcttggagtcctcttcggtccgagccttc<br>tttctttttggaaagtggtcgtctatccgctttccggtaggggccgtcttagcaccctcttcaaccaggccaaaaggttcgagaacccttttcttggcctgaaagactg<br>cctttccgaggtttcccccgaaggatgtgtcgtcggcgagcttctcctgaaactcggcgtccgcgtggttgtacttgaggtaggggttgtctcccgcctcaagctgctc<br>gttgtacgagatgtcgtgctctcgcgcgacctcgtctgccctgttgacaggctctcctcgatcgagaccgtttccgggtccgagatagttataaccaggcagcacaag<br>accacgggcttgatcttgatgctgctgattgggttttggtttcggtgggcccgcttcaaggcccaaaaactcgcgaagaccttcaccaacttcttccaaccaatctgg<br>agggtgatcaacaaaagacat
AAV6	atggctgccgatggttatcttccagattggctcgaggacaacctctctgagggcattcgcgagtggtgggacttgaaacctggagccccgaaacccaaagccaacc<br>agcaaaagcaggacgacggccggggtctggtgcttcctggctacaagtacctcggacccttcaacggactcgacaagggggagcccgtcaacgcggcggatgca<br>gcggccctcgagcacgacaaggcctacgaccagcagctcaaagcgggtgacaatccgtacctgcggtataaccacgccgacgccgagtttcaggagcgtctgca<br>agaagatacgtcttttgggggcaacctcgggcgagcagtcttccaggccaagaagagggttctcgaaccttttggtctggttgaggaaggtgctaagacggctcct<br>ggaaagaaacgtccggtagagcagtcgccacaagagccagactcctcctcgggcattggcaagacaggccagcagcccgctaaaaagagactcaattttggtca<br>gactggcgactcagagtcagtccccgacccacaacctctcggagaacctccagcaacccccgctgctgtgggacctactacaatggcttcaggcggtggcgcacc<br>aatggcagacaataacgaaggcgccgacggagtgggtaatgcctcaggaaattggcattgcgattccacatggctgggcgacagagtcatcaccaccagcaccc<br>gaacatgggccttgcccacctataacaaccacctctacaagcaaatctccagtgcttcaacgggggccagcaacgacaaccactacttcggctacagcaccccctg<br>gggtattttgatttcaacagattccactgccatttctcaccacgtgactggcagcgactcatcaacaacaattggggattccggcccaagagactcaacttcaagc<br>tcttcaacatccaagtcaaggaggtcacgacgaatgatggcgtcacgaccatcgctaataaccttaccagcacggttcaagtcttctcggactcggagtaccagttg<br>ccgtacgtcctcggctctgcgcaccagggctgcctccctccgttcccggcggacgtgttcatgattccgcagtacggctacctaacgctcaacaatggcagccaggc<br>agtgggacggtcatccttttactgcctggaatatttcccatcgcagatgctgagaacgggcaataactttaccttcagctacaccttcgaggacgtgcctttccacag<br>cagctacgcgcacagccagagcctggaccggctgatgaatcctctcatcgaccagtacctgtattacctgaacagaactcagaatcagtccggaagtgcccaaaa<br>caaggacttgctgtttagccgggggtctccagctggcatgtctgttcagcccaaaaactggctacctggaccctgttaccggcagcagcgcgtttctaaaacaaaaa<br>cagacaacaacaacagcaactttacctggactggtgcttcaaaatataaccttaatgggcgtgaatctataatcaaccctggcactgctatggcctcacacaaaga<br>cgacaaagacaagttctttcccatgagcggtgtcatgatttttggaaaggagagcgccggagcttcaaacactgcattggacaatgtcatgatcacagacgaaga<br>ggaaatcaaagccactaaccccgtggccaccgaaagatttgggactgtggcagtcaatctccagagcagcagcacagaccctgcgaccggagatgtgcatgttat<br>gggagccttacctggaatggtgtggcaagacagagacgtatacctgcagggtcctatttggccaaaattcctcacacggatggacactttcacccgtctcctctca<br>tgggcggctttggacttaagcacccgcctcctcagatcctcatcaaaaacacgcctgttcctgcgaatcctccggcagagttttcggctacaaagtttgcttcattcat

	cacccagtattccacaggacaagtgagcgtggagattgaatgggagctgcagaaagaaaacagcaaacgctggaatcccgaagtgcagtatacatctaactatg<br>caaaatctgccaacgttgatttcactgtggacaacaatggactttatactgagcctcgccccattggcacccgttacctcacccgtcccctgtaa
AAV7	atggctgccgatggttatcttccagattggctcgaggacaacctctctgagggcattcgcgagtggtgggacctgaaacctggagccccgaaacccaaagccaacc<br>agcaaaagcaggacaacggccggggtctggtgcttcctggctacaagtacctcggacccttcaacggactcgacaagggggagcccgtcaacgcggcggacgca<br>gcggccctcgagcacgacaaggcctacgaccagcagctcaaagcgggtgacaatccgtacctgcggtataaccacgccgacgccgagtttcaggagcgtctgca<br>agaagatacgtcatttgggggcaacctcgggcgagcagtcttccaggccaagaagcgggttctcgaacctctcggtctggttgaggaaggcgctaagacggctcct<br>gcaaagaagagaccggtagagccgtcacctcagcgttcccccgactcctccacgggcatcggcaagaaaggccagcagcccgccagaaagagactcaatttcgg<br>tcagactggcgactcagagtcagtccccgaccctcaacctctcggagaacctccagcagcgccctctagtgtgggatctggtacagtggctgcaggcggtggcgca<br>ccaatggcagacaataacgaaggtgccgacggagtgggtaatgcctcaggaaattggcattgcgattccacatggctgggcgacagagtcattaccaccagcacc<br>cgaacctgggccctgcccacctacaacaaccacctctacaagcaaatctccagtgaaactgcaggtagtaccaacgacaacacctacttcggctacagcaccccc<br>tgggggtattttgactttaacagattccactgccacttctcaccacgtgactggcagcgactcatcaacaacaactggggattccggcccaagaagctgcggttcaa<br>gctcttcaacatccaggtcaaggaggtcacgacgaatgacggcgttacgaccatcgctaataaccttaccagcacgattcaggtattctcggactcggaataccag<br>ctgccgtacgtcctcggctctgcgcaccagggctgcctgcctccgttcccggcggacgtcttcatgattcctcagtacggctacctgactctcaacaatggcagtcag<br>tctgtgggacgttcctccttctactgcctggagtacttcccctctcagatgctgagaacgggcaacaactttgagttcagctacagcttcgaggacgtgcctttccaca<br>gcagctacgcacacagccagagcctggaccggctgatgaatcccctcatcgaccagtacttgtactacctggccagaacacagagtaacccaggaggcacagctg<br>gcaatcgggaactgcagttttaccagggcgggccttcaactatggccgaacaagccaagaattggttacctggaccttgcttccggcaacaaagagtctccaaaac<br>gctggatcaaaacaacaacagcaactttgcttggactggtgccaccaaatatcacctgaacggcagaaactcgttggttaatcccggcgtcgccatggcaactcac<br>aaggacgacgaggaccgctttttcccatccagcggagtcctgatttttggaaaaactggagcaactaacaaaactacattggaaaatgtgttaatgacaaatgaa<br>gaagaaattcgtcctactaatcctgtagccacggaagaatacgggatagtcagcagcaacttacaagcggctaatactgcagcccagacacaagttgtcaacaac<br>cagggagccttacctggcatggtctggcagaaccgggacgtgtacctgcagggtcccatctgggccaagattcctcacacggatggcaactttcacccgtctcctttt<br>gatgggcggctttggacttaaacatccgcctcctcagatcctgatcaagaacactcccgttcccgctaatcctccggaggtgtttactcctgccaagtttgcttcgttc<br>atcacacagtacagcaccggacaagtcagcgtggaaatcgagtgggagctgcagaaggaaaacagcaagcgctggaacccggagattcagtacacctccaact<br>ttgaaaagcagactggtgtggactttgccgttgacagccagggtgtttactctgagcctcgccctattggcactcgttacctcacccgtaatctgtaa
AAV8	atggctgccgatggttatcttccagattggctcgaggacaacctctctgagggcattcgcgagtggtgggcgctgaaacctggagccccgaagcccaaagccaacc<br>agcaaaagcaggacgacggccggggtctggtgcttcctggctacaagtacctcggacccttcaacggactcgacaagggggagcccgtcaacgcggcggacgca<br>gcggccctcgagcacgacaaggcctacgaccagcagctgcaggcgggtgacaatccgtacctgcggtataaccacgccgacgccgagtttcaggagcgtctgca<br>agaagatacgtcttttgggggcaacctcgggcgagcagtcttccaggccaagaagcgggttctcgaacctctcggtctggttgaggaaggcgctaagacggctcct<br>ggaaagaagagaccggtagagccatcaccccagcgttctccagactcctctacgggcatcggcaagaaaggccaacagcccgccagaaaaagactcaattttgg<br>tcagactggcgactcagagtcagttccagaccctcaacctctcggagaacctccagcagcgccctctggtgtgggacctaatacaatggctgcaggcggtggcgca<br>ccaatggcagacaataacgaaggcgccgacggagtgggtagttcctcgggaaattggcattgcgattccacatggctgggcgacagagtcatcaccaccagcac<br>ccgaacctgggccctgcccacctacaacaaccacctctacaagcaaatctccaacgggacatcgggaggagccaccaacgacaacacctacttcggctacagca

	cccccctgggggtattttgactttaacagattccactgccacttttcaccacgtgactggcagcgactcatcaacaacaactggggattccggcccaagagactcagc<br>ttcaagctcttcaacatccaggtcaaggaggtcacgcagaatgaaggcaccaagaccatcgccaataacctcaccagcaccatccaggtgtttacggactcggag<br>taccagctgccgtacgttctcggctctgcccaccagggctgcctgcctccgttcccggcggacgtgttcatgattccccagtacggctacctaacactcaacaacggt<br>agtcaggccgtgggacgctcctccttctactgcctggaatactttccttcgcagatgctgagaaccggcaacaacttccagtttacttacaccttcgaggacgtgcctt<br>tccacagcagctacgcccacagccagagcttggaccggctgatgaatcctctgattgaccagtacctgtactacttgtctcggactcaaacaacaggaggcacggc<br>aaatacgcagactctgggcttcagccaaggtgggcctaatacaatggccaatcaggcaaagaactggctgccaggaccctgttaccgccaacaacgcgtctcaac<br>gacaaccgggcaaaacaacaatagcaactttgcctggactgctgggaccaaataccatctgaatggaagaaattcattggctaatcctggcatcgctatggcaac<br>acacaaagacgacgaggagcgtttttttcccagtaacgggatcctgatttttggcaaacaaaatgctgccagagacaatgcggattacagcgatgtcatgctcacc<br>agcgaggaagaaatcaaaaccactaaccctgtggctacagaggaatacggtatcgtggcagataacttgcagcagcaaaacacggctcctcaaattggaactgt<br>caacagccagggggccttacccggtatggtctggcagaaccgggacgtgtacctgcagggtcccatctgggccaagattcctcacacggacggcaacttccaccc<br>gtctccgctgatgggcggctttggcctgaaacatcctccgcctcagatcctgatcaagaacacgcctgtacctgcggatcctccgaccaccttcaaccagtcaaagc<br>tgaactctttcatcacgcaatacagcaccggacaggtcagcgtggaaattgaatgggagctgcagaaggaaaacagcaagcgctggaaccccgagatccagtac<br>acctccaactactacaaatctacaagtgtggactttgctgttaatacagaaggcgtgtactctgaaccccgccccattggcacccgttacctcacccgtaatctgtaa
AAV9	atggctgccgatggttatcttccagattggctcgaggacaaccttagtgaaggaattcgcgagtggtgggctttgaaacctggagcccctcaacccaaggcaaatc<br>aacaacatcaagacaacgctcgaggtcttgtgcttccgggttacaaataccttggacccggcaacggactcgacaagggggagccggtcaacgcagcagacgcg<br>gcggccctcgagcacgacaaggcctacgaccagcagctcaaggccggagacaacccgtacctcaagtacaaccacgccgacgccgagttccaggagcggctca<br>aagaagatacgtcttttgggggcaacctcgggcgagcagtcttccaggccaaaaagaggcttcttgaacctcttggtctggttgaggaagcggctaagacggctcc<br>tggaaagaagaggcctgtagagcagtctcctcaggaaccggactcctccgcgggtattggcaaatcgggtgcacagcccgctaaaaagagactcaatttcggtca<br>gactggcgacacagagtcagtcccagaccctcaaccaatcggagaacctcccgcagccccctcaggtgtgggatctcttacaatggcttcaggtggtggcgcacca<br>gtggcagacaataacgaaggtgccgatggagtgggtagttcctcgggaaattggcattgcgattcccaatggctgggggacagagtcatcaccaccagcacccga<br>acctgggccctgcccacctacaacaatcacctctacaagcaaatctccaacagcacatctggaggatcttcaaatgacaacgcctacttcggctacagcaccccct<br>gggggtattttgacttcaacagattccactgccacttctcaccacgtgactggcagcgactcatcaacaacaactggggattccggcctaagcgactcaacttcaag<br>ctcttcaacattcaggtcaaagaggttacggacaacaatggagtcaagaccatcgccaataaccttaccagcacggtccaggtcttcacggactcagactatcagc<br>tcccgtacgtgctcgggtcggctcacgagggctgcctcccgccgttcccagcggacgttttcatgattcctcagtacgggtatctgacgcttaatgatggaagccagg<br>ccgtgggtcgttcgtccttttactgcctggaatatttcccgtcgcaaatgctaagaacgggtaacaacttccagttcagctacgagtttgagaacgtacctttccatag<br>cagctacgctcacagccaaagcctggaccgactaatgaatccactcatcgaccaatacttgtactatctctcaaagactattaacggttctggacagaatcaacaa<br>acgctaaaattcagtgtggccggacccagcaacatggctgtccagggaagaaactacatacctggacccagctaccgacaacaacgtgtctcaaccactgtgact<br>caaaacaacaacagcgaatttgcttggcctggagcttcttcttgggctctcaatggacgtaatagcttgatgaatcctggacctgctatggccagccacaaagaag<br>gagaggaccgtttctttcctttgtctggatctttaatttttggcaaacaaggaactggaagagacaacgtggatgcggacaaagtcatgataaccaacgaagaaga<br>aattaaaactactaacccggtagcaacggagtcctatggacaagtggccacaaaccaccagagtgcccaagcacaggcgcagaccggctgggttcaaaaccaa<br>ggaatacttccgggtatggtttggcaggacagagatgtgtacctgcaaggacccatttgggccaaaattcctcacacggacggcaactttcacccttctccgctgat

	gggagggtttggaatgaagcacccgcctcctcagatcctcatcaaaaacacacctgtacctgcggatcctccaacggccttcaacaaggacaagctgaactctttc<br>atcacccagtattctactggccaagtcagcgtggagatcgagtgggagctgcagaaggaaaacagcaagcgctggaacccggagatccagtacacttccaactat<br>tacaagtctaataatgttgaatttgctgttaatactgaaggtgtatatagtgaaccccgccccattggcaccagatacctgactcgtaatctgtaa
DJ8	atggctgccgatggttatcttccagattggctcgaggacactctctctgaaggaataagacagtggtggaagctcaaacctggcccaccaccaccaaagcccgcag<br>agcggcataaggacgacagcaggggtcttgtgcttcctgggtacaagtacctcggacccttcaacggactcgacaagggagagccggtcaacgaggcagacgcc<br>gcggccctcgagcacgacaaagcctacgaccggcagctcgacagcggagacaacccgtacctcaagtacaaccacgccgacgccgagttccaggagcggctca<br>aagaagatacgtcttttgggggcaacctcgggcgagcagtcttccaggccaaaaagaggcttcttgaacctcttggtctggttgaggaagcggctaagacggctcc<br>tggaaagaagaggcctgtagagcactctcctgtggagccagactcctcctcgggaaccggaaaggcgggccagcagcctgcaagaaaaagattgaattttggtc<br>agactggagacgcagactcagtcccagaccctcaaccaatcggagaacctcccgcagccccctcaggtgtgggatctcttacaatggctgcaggcggtggcgcac<br>caatggcagacaataacgagggcgccgacggagtgggtaattcctcgggaaattggcattgcgattccacatggatgggcgacagagtcatcaccaccagcacc<br>cgaacctgggccctgcccacctacaacaaccacctctacaagcaaatctccaacagcacatctggaggatcttcaaatgacaacgcctacttcggctacagcaccc<br>cctgggggtattttgactttaacagattccactgccacttttcaccacgtgactggcagcgactcatcaacaacaactggggattccggcccaagagactcagcttc<br>aagctcttcaacatccaggtcaaggaggtcacgcagaatgaaggcaccaagaccatcgccaataacctcaccagcaccatccaggtgtttacggactcggagtac<br>cagctgccgtacgttctcggctctgcccaccagggctgcctgcctccgttcccggcggacgtgttcatgattccccagtacggctacctaacactcaacaacggtagt<br>caggccgtgggacgctcctccttctactgcctggaatactttccttcgcagatgctgagaaccggcaacaacttccagtttacttacaccttcgaggacgtgcctttcc<br>acagcagctacgcccacagccagagcttggaccggctgatgaatcctctgattgaccagtacctgtactacttgtctcggactcaaacaacaggaggcacgacaa<br>atacgcagactctgggcttcagccaaggtgggcctaatacaatggccaatcaggcaaagaactggctgccaggaccctgttaccgccagcagcgagtatcaaag<br>acatctgcggataacaacaacagtgaatactcgtggactggagctaccaagtaccacctcaatggcagagactctctggtgaatccgggcccggccatggcaagc<br>cacaaggacgatgaagaaaagtttttcctcagagcggggttctcatctttgggaagcaaggctcagagaaaacaaatgtggacattgaaaaggtcatgattaca<br>gacgaagaggaaatcaggacaaccaatcccgtggctacggagcagtatggttctgtatctaccaacctccagcaaggcaacacacaagcagctaccgcagatgt<br>caacacacaaggcgttcttccaggcatggtctggcaggacagagatgtgtaccttcaggggcccatctgggcaaagattccacacacggacggacattttcacccc<br>tctcccctcatgggtggattcggacttaaacaccctccgcctcagatcctgatcaagaacacgcctgtacctgcggatcctccgaccaccttcaaccagtcaaagct<br>gaactctttcatcacccagtattctactggccaagtcagcgtggagatcgagtgggagctgcagaaggaaaacagcaagcgctggaaccccgagatccagtacac<br>ctccaactactacaaatctacaagtgtggactttgctgttaatacagaaggcgtgtactctgaaccccgccccattggcacccgttacctcacccgtaatctgtaa
DJ N589X	atggctgccgatggttatcttccagattggctcgaggacactctctctgaaggaataagacagtggtggaagctcaaacctggcccaccaccaccaaagcccgcag<br>agcggcataaggacgacagcaggggtcttgtgcttcctgggtacaagtacctcggacccttcaacggactcgacaagggagagccggtcaacgaggcagacgcc<br>gcggccctcgagcacgacaaagcctacgaccggcagctcgacagcggagacaacccgtacctcaagtacaaccacgccgacgccgagttccaggagcggctca<br>aagaagatacgtcttttgggggcaacctcgggcgagcagtcttccaggccaaaaagaggcttcttgaacctcttggtctggttgaggaagcggctaagacggctcc<br>tggaaagaagaggcctgtagagcactctcctgtggagccagactcctcctcgggaaccggaaaggcgggccagcagcctgcaagaaaaagattgaattttggtc<br>agactggagacgcagactcagtcccagaccctcaaccaatcggagaacctcccgcagccccctcaggtgtgggatctcttacaatggctgcaggcggtggcgcac<br>caatggcagacaataacgagggcgccgacggagtgggtaattcctcgggaaattggcattgcgattccacatggatgggcgacagagtcatcaccaccagcacc

	cgaacctgggccctgcccacctacaacaaccacctctacaagcaaatctccaacagcacatctggaggatcttcaaatgacaacgcctacttcggctacagcaccc<br>cctgggggtattttgactttaacagattccactgccacttttcaccacgtgactggcagcgactcatcaacaacaactggggattccggcccaagagactcagcttc<br>aagctcttcaacatccaggtcaaggaggtcacgcagaatgaaggcaccaagaccatcgccaataacctcaccagcaccatccaggtgtttacggactcggagtac<br>cagctgccgtacgttctcggctctgcccaccagggctgcctgcctccgttcccggcggacgtgttcatgattccccagtacggctacctaacactcaacaacggtagt<br>caggccgtgggacgctcctccttctactgcctggaatactttccttcgcagatgctgagaaccggcaacaacttccagtttacttacaccttcgaggacgtgcctttcc<br>acagcagctacgcccacagccagagcttggaccggctgatgaatcctctgattgaccagtacctgtactacttgtctcggactcaaacaacaggaggcacgacaa<br>atacgcagactctgggcttcagccaaggtgggcctaatacaatggccaatcaggcaaagaactggctgccaggaccctgttaccgccagcagcgagtatcaaag<br>acatctgcggataacaacaacagtgaatactcgtggactggagctaccaagtaccacctcaatggcagagactctctggtgaatccgggcccggccatggcaagc<br>cacaaggacgatgaagaaaagtttttcctcagagcggggttctcatctttgggaagcaaggctcagagaaaacaaatgtggacattgaaaaggtcatgattaca<br>gacgaagaggaaatcaggacaaccaatcccgtggctacggagcagtatggttctgtatctaccaacctccagagaggctag
AAV2-retro	atggctgccgatggttatcttccagattggctcgaggacactctctctgaaggaataagacagtggtggaagctcaaacctggcccaccaccaccaaagcccgcag<br>agcggcataaggacgacagcaggggtcttgtgcttcctgggtacaagtacctcggacccttcaacggactcgacaagggagagccggtcaacgaggcagacgcc<br>gcggccctcgagcacgacaaagcctacgaccggcagctcgacagcggagacaacccgtacctcaagtacaaccacgccgacgcggagtttcaggagcgccttaa<br>agaagatacgtcttttgggggcaacctcggacgagcagtcttccaggcgaaaaagagggttcttgaacctctgggcctggttgaggaacctgttaagacggctccg<br>ggaaaaaagaggccggtagagcactctcctgtggagccagactcctcctcgggaaccggaaaggcgggccagcagcctgcaagaaaaagattgaattttggtca<br>gactggagacgcagactcagtacctgacccccagcctctcggacagccaccagcagccccctctggtctgggaactaatacgatggctacaggcagtggcgcacc<br>aatggcagacaataacgagggcgccgacggagtgggtaattcctcgggaaattggcattgcgattccacatggatgggcgacagagtcatcaccaccagcaccc<br>gaacctgggccctgcccacctacaacaaccacctctacaaacaaatttccagccaatcaggagcctcgaacgacaatcactactttggctacagcaccccttgggg<br>gtattttgacttcaacagattccactgccacttttcaccacgtgactggcaaagactcatcaacaacaactggggattccgacccaagagactcaacttcaagctctt<br>taacattcaagtcaaagaggtcacgcagaatgacggtacgacgacgattgccaataaccttaccagcacggttcaggtgtttactgactcggagtaccagctcccg<br>tacgtcctcggctcggcgcatcaaggatgcctcccgccgttcccagcagacgtcttcatggtgccacagtatggatacctcaccctgaacgacgggagtcaggcag<br>taggacgctcttcattttactgcctggagtactttccttctcagatgctgcgtaccggaaacaactttaccttcagctacacttttgaggacgttcctttccacagcagc<br>tacgctcacagccagagtctggaccgtctcatgaatcctctcatcgaccagtacctgtattacttgagcagaacaaacactccaagtggaaccaccacgcagtcaa<br>ggcttcagttttctcaggccggagcgagtgacattcgggaccagtctaggaactggcttcctggaccctgttaccgccagcagcgagtatcaaagacatctgcggat<br>aacaacaacagtgaatactcgtggactggagctaccaagtaccacctcaatggcagagactctctggtgaatccgggcccggccatggcaagccacaaggacga<br>tgaagaaaagtttttcctcagagcggggttctcatctttgggaagcaaggctcagagaaaacaaatgtggacattgaaaaggtcatgattacagacgaagagga<br>aatcaggacaaccaatcccgtggctacggagcagtatggttctgtatctaccaacctccagagaggcaacctagcagaccaagactacacaaaaactgctaggc<br>aagcagctaccgcagatgtcaacacacaaggcgttcttccaggcatggtctggcaggacagagatgtgtaccttcaggggcccatctgggcaaagattccacaca<br>cggacggacattttcacccctctcccctcatgggtggattcggacttaaacaccctcctccccagattctcatcaagaacaccccggtacctgcgaatccttcgacca<br>ccttcagtgcggcaaagtttgcttccttcatcacacagtactccacgggacaggtcagcgtggagatcgagtgggagctgcagaaggaaaacagcaaacgctgga

	atcccgaaattcagtacacttccaactacaacaagtctattaatgtggactttactgtggacactaatggcgtgtattcagagcctcgccccattggcaccagatacc<br>tgactcgtaatctgtaa
PHP.S	atggctgccgatggttatcttccagattggctcgaggacaaccttagtgaaggaattcgcgagtggtgggctttgaaacctggagcccctcaacccaaggcaaatc<br>aacaacatcaagacaacgctagaggtcttgtgcttccgggttacaaataccttggacccggcaacggactcgacaagggggagccggtcaacgcagcagacgcg<br>gcggccctcgagcacgacaaagcctacgaccagcagctcaaggccggagacaacccgtacctcaagtacaaccacgccgacgccgagttccaggagcggctca<br>aagaagatacgtcttttgggggcaacctcgggcgagcagtcttccaggccaaaaagaggcttcttgaacctcttggtctggttgaggaagcggctaagacggctcc<br>tggaaagaagaggcctgtagagcagtctcctcaggaaccggactcctccgcgggtattggcaaatcgggtgcacagcccgctaaaaagagactcaatttcggtca<br>gactggcgacacagagtcagtcccagaccctcaaccaatcggagaacctcccgcagccccctcaggtgtgggatctcttacaatggcttcaggtggtggcgcacca<br>gtggcagacaataacgaaggtgccgatggagtgggtagttcctcgggaaattggcattgcgattcccaatggctgggggacagagtcatcaccaccagcacccga<br>acctgggccctgcccacctacaacaatcacctctacaagcaaatctccaacagcacatctggaggatcttcaaatgacaacgcctacttcggctacagcaccccct<br>gggggtattttgacttcaacagattccactgccacttctcaccacgtgactggcagcgactcatcaacaacaactggggattccggcctaagcgactcaacttcaag<br>ctctttaacattcaggtcaaagaggttacggacaacaatggagtcaagaccatcgccaataaccttaccagcacggtccaggtcttcacggactcagactatcagc<br>tcccgtacgtgctcgggtcggctcacgagggctgcctcccgccgttcccagcggacgttttcatgattcctcagtacgggtatctgacgcttaatgatggaagccagg<br>ccgtgggtcgttcgtccttttactgcctggaatatttcccgtcgcaaatgctaagaacgggtaacaacttccagttcagctacgagtttgagaacgtacctttccatag<br>cagctacgctcacagccaaagcctggaccgactaatgaatccactcatcgaccaatacttgtactatctctctagaactattaacggttctggacagaatcaacaaa<br>cgctaaaattcagtgtggccggacccagcaacatggctgtccagggaagaaactacatacctggacccagctaccgacaacaacgtgtctcaaccactgtgactc<br>aaaacaacaacagcgaatttgcttggcctggagcttcttcttgggctctcaatggacgtaatagcttgatgaatcctggacctgctatggcctctcacaaagaagga<br>gaggaccgtttctttcctttgtctggatctttaatttttggcaaacaaggtactggcagagacaacgtggatgcggacaaagtcatgataaccaacgaagaagaaat<br>taaaactactaacccggtagcaacggagtcctatggacaagtggccacaaaccaccagagtgcccaacaggcggttaggacgtctttggcacaggcgcagaccg<br>gttgggttcaaaaccaaggaatacttccgggtatggtttggcaggacagagatgtgtacctgcaaggacccatttgggccaaaattcctcacacggacggcaactt<br>tcacccttctccgctgatgggagggtttggaatgaagcacccgcctcctcagatcctcatcaaaaacacacctgtacctgcggatcctccaacggccttcaacaagg<br>acaagctgaactctttcatcacccagtattctactggtcaagtcagcgtggagatcgagtgggagctgcagaaggaaaacagcaagcgctggaacccggagatcc<br>agtacacttccaactattacaagtctaataatgttgaatttgctgttaatactgaaggtgtatatagtgaaccccgccccattggcaccagatacctgactcgtaatct<br>gtaa
PHP.eB	atggctgccgatggttatcttccagattggctcgaggacaaccttagtgaaggaattcgcgagtggtgggctttgaaacctggagcccctcaacccaaggcaaatc<br>aacaacatcaagacaacgctagaggtcttgtgcttccgggttacaaataccttggacccggcaacggactcgacaagggggagccggtcaacgcagcagacgcg<br>gcggccctcgagcacgacaaagcctacgaccagcagctcaaggccggagacaacccgtacctcaagtacaaccacgccgacgccgagttccaggagcggctca<br>aagaagatacgtcttttgggggcaacctcgggcgagcagtcttccaggccaaaaagaggcttcttgaacctcttggtctggttgaggaagcggctaagacggctcc<br>tggaaagaagaggcctgtagagcagtctcctcaggaaccggactcctccgcgggtattggcaaatcgggtgcacagcccgctaaaaagagactcaatttcggtca<br>gactggcgacacagagtcagtcccagaccctcaaccaatcggagaacctcccgcagccccctcaggtgtgggatctcttacaatggcttcaggtggtggcgcacca<br>gtggcagacaataacgaaggtgccgatggagtgggtagttcctcgggaaattggcattgcgattcccaatggctgggggacagagtcatcaccaccagcacccga

	acctgggccctgcccacctacaacaatcacctctacaagcaaatctccaacagcacatctggaggatcttcaaatgacaacgcctacttcggctacagcaccccct<br>gggggtattttgacttcaacagattccactgccacttctcaccacgtgactggcagcgactcatcaacaacaactggggattccggcctaagcgactcaacttcaag<br>ctctttaacattcaggtcaaagaggttacggacaacaatggagtcaagaccatcgccaataaccttaccagcacggtccaggtcttcacggactcagactatcagc<br>tcccgtacgtgctcgggtcggctcacgagggctgcctcccgccgttcccagcggacgttttcatgattcctcagtacgggtatctgacgcttaatgatggaagccagg<br>ccgtgggtcgttcgtccttttactgcctggaatatttcccgtcgcaaatgctaagaacgggtaacaacttccagttcagctacgagtttgagaacgtacctttccatag<br>cagctacgctcacagccaaagcctggaccgactaatgaatccactcatcgaccaatacttgtactatctctctagaactattaacggttctggacagaatcaacaaa<br>cgctaaaattcagtgtggccggacccagcaacatggctgtccagggaagaaactacatacctggacccagctaccgacaacaacgtgtctcaaccactgtgactc<br>aaaacaacaacagcgaatttgcttggcctggagcttcttcttgggctctcaatggacgtaatagcttgatgaatcctggacctgctatggcctctcacaaagaagga<br>gaggaccgtttctttcctttgtctggatctttaatttttggcaaacaaggtactggcagagacaacgtggatgcggacaaagtcatgataaccaacgaagaagaaat<br>taaaactactaacccggtagcaacggagtcctatggacaagtggccacaaaccaccagagtgatgggactttggcggtgccttttaaggcacaggcgcagaccgg<br>ttgggttcaaaaccaaggaatacttccgggtatggtttggcaggacagagatgtgtacctgcaaggacccatttgggccaaaattcctcacacggacggcaacttt<br>cacccttctccgctgatgggagggtttggaatgaagcacccgcctcctcagatcctcatcaaaaacacacctgtacctgcggatcctccaacggccttcaacaagg<br>acaagctgaactctttcatcacccagtattctactggtcaagtcagcgtggagatcgagtgggagctgcagaaggaaaacagcaagcgctggaacccggagatcc<br>agtacacttccaactattacaagtctaataatgttgaatttgctgttaatactgaaggtgtatatagtgaaccccgccccattggcaccagatacctgactcgtaatct<br>gtaa
Sch9	atggctgccgatggttatcttccagattggctcgaggacaacctctctgagggcattcgcgagtggtgggacctgaaacctggagccccgaaacccaaagccaacc<br>agcaaaagcaggacgacggccggggtctggtgcttcctggctacaagtacctcggacccttcaacggactcgacaagggggagcccgtcaacgcggcggatgca<br>gcggccctcgaacacgacaaggcctacgaccagcagctcaaagcgggtgacaatccgtacctgcggtataaccacgccgacgccgagtttcaggagcgtctgca<br>agaagatacgtcttttgggggcaacctcgggcgagcagtcttccaggccaagaagagggttctcgaacctcttggtctggttgaggaagcggctaagacggctcct<br>ggaaagaagaggcctgtagagcagtctcctcaggaaccggactcctccgcgggtattggcaaatcgggtgcacagcccgctaaaaagagactcaatttcggtcag<br>actggcgacacagagtcagtcccagaccctcaaccaatcggagaacctcccgcagccccctcaggtgtgggatctcttacaatggcttcaggtggtggcgcaccag<br>tggcagacaataacgaaggtgccgatggagtgggtagttcctcgggaaattggcattgcgattcccaatggctgggggacagagtcatcaccaccagcacccgaa<br>cctgggccctgcccacctacaacaatcacctctacaagcaaatctccaacagcacatctggaggatcttcaaatgacaacgcctacttcggctacagcaccccctg<br>ggggtattttgactttaacagattccactgccacttttcaccacgtgactggcagcgactcatcaacaacaactggggattccggcccaagagactcagcttcaagc<br>tcttcaacatccaggtcaaggaggtcacgcagaatgaaggcaccaagaccatcgccaataacctcaccagcaccatccaggtctttacggactcagactatcagc<br>tcccgtacgtgctcgggtcggctcacgagggctgcctcccgccgttcccagcggacgttttcatgattcctcagtacgggtatctgacgcttaatgatggaagccagg<br>ccgtgggtcgttcgtccttttactgcctggaatatttcccgtcgcaaatgctaagaacgggtaacaacttccagttcagctacgagtttgagaacgtacctttccatag<br>cagctacgctcacagccaaagcctggaccgactaatgaatccactcatcgaccaatacttgtactatctctcaaagactattaacggttctggacagaatcaacaa<br>acgctaaaattcagtgtggccggacccagcaacatggctgtccagggaagaaactggcttcctggaccctgttaccgccagcagcgagtatcaaagacatctgcg<br>gataacaacaacagtgaatactcgtggactggagctaccaagtaccacctcaatggcagagactctctggtgaatccgggcccggccatggcaagccacaagga<br>cgatgaagaaaagtttttcctcagagcggggttctcatctttgggaagcaaggctcagagaaaacaaatgtggacattgaaaaggtcatgattacagacgaaga

	ggaaatcaggacaaccaatcccgtggctacggagcagtatggttctgtatctaccaacctccagagaggcaacagacaagcagctaccgcagatgtcaacacac<br>aaggcgttcttccaggcatggtctggcaggacagagacgtgtacctgcaaggacccatttgggccaaaattcctcacacggacggcaactttcacccttctccgctg<br>atgggagggtttggaatgaagcacccgcctcctcagatcctcatcaaaaacacacctgtacctgcggatcctccaacggccttcaacaaggacaagctgaactctt<br>tcatcacccagtattctactggccaagtcagcgtggagatcgagtgggagctgcagaaggaaaacagcaagcgctggaacccggagatccagtacacttccaact<br>attacaagtctaataatgttgaatttgctgttaatactgaaggtgtatatagtgaaccccgccccattggcaccagatacctgactcgtaatctgtaa
rh10	atggctgccgatggttatcttccagattggctcgaggacaacctctctgagggcattcgcgagtggtgggacttgaaacctggagccccgaaacccaaagccaacc<br>agcaaaagcaggacgacggccggggtctggtgcttcctggctacaagtacctcggacccttcaacggactcgacaagggggagcccgtcaacgcggcggacgca<br>gcggccctcgagcacgacaaggcctacgaccagcagctcaaagcgggtgacaatccgtacctgcggtataaccacgccgacgccgagtttcaggagcgtctgca<br>agaagatacgtcttttgggggcaacctcgggcgagcagtcttccaggccaagaagcgggttctcgaacctctcggtctggttgaggaaggcgctaagacggctcct<br>ggaaagaagagaccggtagagccatcaccccagcgttctccagactcctctacgggcatcggcaagaaaggccagcagcccgcgaaaaagagactcaactttg<br>ggcagactggcgactcagagtcagtgcccgaccctcaaccaatcggagaaccccccgcaggcccctctggtctgggatctggtacaatggctgcaggcggtggcg<br>ctccaatggcagacaataacgaaggcgccgacggagtgggtagttcctcaggaaattggcattgcgattccacatggctgggcgacagagtcatcaccaccagca<br>cccgaacctgggccctccccacctacaacaaccacctctacaagcaaatctccaacgggacttcgggaggaagcaccaacgacaacacctacttcggctacagca<br>ccccctgggggtattttgactttaacagattccactgccacttctcaccacgtgactggcagcgactcatcaacaacaactggggattccggcccaagagactcaac<br>ttcaagctcttcaacatccaggtcaaggaggtcacgcagaatgaaggcaccaagaccatcgccaataaccttaccagcacgattcaggtctttacggactcggaat<br>accagctcccgtacgtcctcggctctgcgcaccagggctgcctgcctccgttcccggcggacgtcttcatgattcctcagtacgggtacctgactctgaacaatggca<br>gtcaggccgtgggccgttcctccttctactgcctggagtactttccttctcaaatgctgagaacgggcaacaactttgagttcagctaccagtttgaggacgtgcctttt<br>tcacagcagctacgcgcacagccaaagcctggaccggctgatgaacccccctcatcgaccagtacctgtactacctgtctcggactcagtccacgggaggtaccgca<br>ggaactcagcagttgctattttctcaggccgggcctaataacatgtcggctcaggccaaaaactggctacccgggccctgctaccggcagcaacgcgtctccacga<br>cactgtcgcaaaataacaacagcaactttgcctggaccggtgccaccaagtatcatctgaatggcagagactctctggtaaatcccggtgtcgctatggcaaccca<br>caaggacgacgaagagcgatttttccgtccagcggagtcttaatgtttgggaaacagggagctggaaaagacaacgtggactatagcagcgttatgctaaccag<br>tgaggaagaaattaaaaccaccaacccagtggccacagaacagtacggcgtggtggccgataacctgcaacagcaaaacgccgctcctattgtaggggccgtca<br>acagtcaaggagccttacctggcatggtctggcagaaccgggacgtgtacctgcagggtcctatctgggccaagattcctcacacggacggaaactttcatccctc<br>gccgctgatgggaggctttggactgaaacacccgcctcctcagatcctgattaagaatacacctgttcccgcggatcctccaactaccttcagtcaagctaagctgg<br>cgtcgttcatcacgcagtacagcaccggacaggtcagcgtggaaattgaatgggagctgcagaaagaaaacagcaaacgctggaacccagagattcaatacact<br>tccaactactacaaatctacaaatgtggactttgctgttaacacagatggcacttattctgagcctcgccccatcggcacccgttacctcacccgtaatctgtaa
ITR-CAG-eGFP-WPRE-hGH polyA-ITR sequence	cgcgctcgctcgctcactgaggccgcccgggcaaagcccgggcgtcgggcgacctttggtcgcccggcctcagtgagcgagcgagcgcgcagagagggagtggc<br>caactccatcactaggggttccttgtagttaatgattaacccgccatgctacttatctacgtagccatgctctaggaagagtaccattgacgtcaataatgacgtatg<br>ttcccatagtaacgccaatagggactttccattgacgtcaatgggtggagtatttacggtaaactgcccacttggcagtacatcaagtgtatcatatgccaagtacgc<br>cccctattgacgtcaatgacggtaaatggcccgcctggcattatgcccagtacatgaccttatgggactttcctacttggcagtacatctacgtattagtcatcgctat<br>taccatggtcgaggtgagccccacgttctgcttcactctccccatctcccccccctccccacccccaattttgtatttatttattttttaattattttgtgcagcgatgggg

(modified from Addgene #37825; CAG promoter underlined)	cgggggggggggggggggcgcgccaggcggggcggggcggggcgaggggcggggcggggcgaggcggagaggtgcggcggcagccaatcagagcggcgc<br>gctccgaaagtttccttttatggcgaggcggcggcggcggcggccctataaaaagcgaagcgcgcggcgggcgggagtcgctgcgcgctgccttcgccccgtgcc<br>ccgctccgccgccgcctcgcgccgcccgccccggctctgactgaccgcgttactcccacaggtgagcgggcgggacggcccttctcctccgggctgtaattagcgct<br>tggtttaatgacggcttgtttcttttctgtggctgcgtgaaagccttgaggggctccgggagggccctttgtgcggggggagcggctcggggctgtccgcgggggga<br>cggctgccttcgggggggacggggcagggcggggttcggcttctggcgtgtgaccggcggctctagagcctctgctaaccatgttcatgccttcttctttttcctaca<br>gctcctgggcaacgtgctggttattgtgctgtctcatcattttggcaaagaattggatccgccaccatggtgagcaagggcgaggagctgttcaccggggtggtgcc<br>catcctggtcgagctggacggcgacgtaaacggccacaagttcagcgtgtccggcgagggcgagggcgatgccacctacggcaagctgaccctgaagttcatttg<br>caccaccggcaagctgcccgtgccctggcccaccctcgtgaccaccctgacctacggcgtgcagtgcttcagccgctaccccgaccacatgaagcagcacgacttc<br>ttcaagtccgccatgcccgaaggctacgtccaggagcgcaccatcttcttcaaggacgacggcaactacaagacccgcgccgaggtgaagttcgagggcgacacc<br>ctggtgaaccgcatcgagctgaagggcatcgacttcaaggaggacggcaacatcctggggcacaagctggagtacaactacaacagccacaacgtctatatcat<br>ggccgacaagcagaagaacggcatcaaggtgaacttcaagatccgccacaacatcgaggacggcagcgtgcagctcgccgaccactaccagcagaacacccccc<br>atcggcgacggccccgtgctgctgcccgacaaccactacctgagcacccagtccgccctgagcaaagaccccaacgagaagcgcgatcacatggtcctgctggag<br>ttcgtgaccgccgccgggatcactctcggcatggacgagctgtacaagtaagaattcgatatcaagcttatcgataatcaacctctggattacaaaatttgtgaaag<br>attgactggtattcttaactatgttgctccttttacgctatgtggatacgctgctttaatgcctttgtatcatgctattgcttcccgtatggctttcattttctcctccttgta<br>taaatcctggttgctgtctctttatgaggagttgtggcccgttgtcaggcaacgtggcgtggtgtgcactgtgtttgctgacgcaacccccactggttggggcattgcc<br>accacctgtcagctcctttccgggactttcgctttccccctccctattgccacggcggaactcatcgccgcctgccttgcccgctgctggacaggggctcggctgttgg<br>gcactgacaattccgtggtgttgtcggggaaatcatcgtcctttccttggctgctcgcctgtgttgccacctggattctgcgcgggacgtccttctgctacgtcccttcg<br>gccctcaatccagcggaccttccttcccgcggcctgctgccggctctgcggcctcttccgcgtcttcgccttcgccctcagacgagtcggatctccctttgggccgcct<br>ccccgtatcgataccgtcgacccgggcggccgcttcgagcagacatgagggtggcatccctgtgacccctccccagtgcctctcctggccctggaagttgccactcc<br>agtgcccaccagccttgtcctaataaaattaagttgcatcattttgtctgactaggtgtccttctataatattatggggtggaggggggtggtatggagcaaggggca<br>agttgggaagacaacctgtagggcctgcggggtctattgggaaccaagctggagtgcagtggcacaatcttggctcactgcaatctccgcctcctgggttcaagcg<br>attctcctgcctcagcctcccgagttgttgggattccaggcatgcatgaccaggctcagctaatttttgtttttttggtagagacggggtttcaccatattggccaggct<br>ggtctccaactcctaatctcaggtgatctacccaccttggcctcccaaattgctgggattacaggcgtgaaccactgctcccttccctgtccttaacaacaacaattgc<br>attcattttatgtttcaggttcagggggagatgtgggaggtttttttaaagcaagtaaaacctctacaaatgtggtaaaatcgataaggatcttcctagagcatggcta<br>cgtagataagtagcatggcgggttaatcattaactacaaggaacccctagtgatggagttggccactccctctctgcgcgctcgctcgctcactgaggccgggcgac<br>caaaggtcgcccgacgcccgggctttgcccgggcggcctcagtgagcgagcgagcgcgcagctgcattaatgaatcggccaacgcgcggggagaggcggtttgc<br>gtattgggcgctcttccgcttcctcgctcactgactcgctgcgctcggtcgttcggctgcggcgagcggtatcagctcactcaaaggcggtaatacggttatccacag<br>aatcaggggataacgcaggaaagaacatgtgagcaaaaggccagcaaaaggccaggaaccgtaaaaaggccgcgttgctggcgtttttccataggctccgcccc<br>cctgacgagcatcacaaaaatcgacgctcaagtcagaggtggcgaaacccgacaggactataaagataccaggcgtttccccctggaagctccctcgtgcgctct<br>cctgttccgaccctgccgcttaccggatacctgtccgcctttctcccttcgggaagcgtggcgctttctcatagctcacgctgtaggtatctcagttcggtgtaggtcgt<br>tcgctccaagctgggctgtgtgcacgaaccccccgttcagcccgaccgctgcgccttatccggtaactatcgtcttgagtccaacccggtaagacacgacttatcgc

	cactggcagcagccactggtaacaggattagcagagcgaggtatgtaggcggtgctacagagttcttgaagtggtggcctaactacggctacactagaagaacagtatttggtatctgcgctctgctgaagccagttaccttcggaaaaagagttggtagctcttgatccggcaaacaaaccaccgctggtagcggtggtttttttgtttgcaagcagcagattacgcgcagaaaaaaaggatctcaagaagatcctttgatcttttctacggggtctgacgctcagtggaacgaaaactcacgttaagggattttggtcatgagattatcaaaaaggatcttcacctagatccttttaaattaaaaatgaagttttaaatcaatctaaagtatatatgagtaaacttggtctgacagttaccaatgcttaatcagtgaggcacctatctcagcgatctgtctatttcgttcatccatagttgcctgactccccgtcgtgtagataactacgatacgggagggcttaccatctggccccagtgctgcaatgataccgcgagacccacgctcaccggctccagatttatcagcaataaaccagccagccggaagggccgagcgcagaagtggtcctgcaactttatccgcctccatccagtctattaattgttgccgggaagctagagtaagtagttcgccagttaatagtttgcgcaacgttgttgccattgctacaggcatcgtggtgtcacgctcgtcgtttggtatggcttcattcagctccggttcccaacgatcaaggcgagttacatgatcccccatgttgtgcaaaaaagcggttagctccttcggtcctccgatcgttgtcagaagtaagttggccgcagtgttatcactcatggttatggcagcactgcataattctcttactgtcatgccatccgtaagatgcttttctgtgactggtgagtactcaaccaagtcattctgagaatagtgtatgcggcgaccgagttgctcttgcccggcgtcaatacgggataataccgcgccacatagcagaactttaaaagtgctcatcattggaaaacgttcttcggggcgaaaactctcaaggatcttaccgctgttgagatccagttcgatgtaacccactcgtgcacccaactgatcttcagcatcttttactttcaccagcgtttctgggtgagcaaaaacaggaaggcaaaatgccgcaaaaaagggaataagggcgacacggaaatgttgaatactcatactcttcctttttcaatattattgaagcatttatcagggttattgtctcatgagcggatacatatttgaatgtatttagaaaaataaacaaataggggttccgcgcacatttccccgaaaagtgccacctaaattgtaagcgttaatattttgttaaaattcgcgttaaatttttgttaaatcagctcattttttaaccaataggccgaaatcggcaaaatcccttataaatcaaaagaatagaccgagatagggttgagtgttgttccagtttggaacaagagtccactattaaagaacgtggactccaacgtcaaagggcgaaaaaccgtctatcagggcgatggcccactacgtgaaccatcaccctaatcaagttttttggggtcgaggtgccgtaaagcactaaatcggaaccctaaagggagcccccgatttagagcttgacggggaaagccggcgaacgtggcgagaaaggaagggaagaaagcgaaaggagcgggcgctagggcgctggcaagtgtagcggtcacgctgcgcgtaaccaccacacccgccgcgcttaatgcgccgctacagggcgcgtcccattcgccattcaggctgcgcaactgttgggaagggcgatcggtgcgggcctcttcgctattacgccagctg
**AAV1 BC**	CGACGGTCGTTTACCTTGTGGATCA
**AAV2 BC**	CGACGAGTCGTTTACCTTGTGGATC
**AAV5 BC**	CGACGGGATCAGTCGTTTACCTTGT
**AAV6 BC**	CGACGTTACCTTGTGGATCAGTCGT
**AAV7 BC**	CGACGGTTTACCTTGTGGATCAGTC
**AAV8 BC**	CGACGCGTTTACCTTGTGGATCAGT
**AAV9 BC**	CGACGACCTTGTGGATCAGTCGTTT
**DJ8 BC**	CGACGTGGATCAGTCGTTTACCTTG
**DJ BC**	CGACGTTTACCTTGTGGATCAGTCG
**2retro BC**	CGACGCCTTGTGGATCAGTCGTTTA
**PHP.s BC**	CGACGTCAGTCGTTTACCTTGTGGA

**PHP.eB BC**	CGACGGATCAGTCGTTTACCTTGTG
**sch9 BC**	CGACGTCGTTTACCTTGTGGATCAG
**rh10 BC**	CGACGCAGTCGTTTACCTTGTGGAT

bioRxiv preprint doi: https://doi.org/10.1101/2024.11.14.623624; this version posted November 15, 2024. The copyright holder for this preprint (which was not certified by peer review) is the author/funder, who has granted bioRxiv a license to display the preprint in perpetuity. It is made available under aCC-BY 4.0 International license.

Supplemental Table 2 | Estimates of intraparenchymal trial viral dosing per mg of brain tissue. Using published estimates of diffusion spread during convection-enhanced delivery and the mass of brain tissue, we calculated the estimated range of vector genomes per mg of brain tissue dosed in intraparenchymal trials for which data could be found, assuming 1-3 µLof brain infused per µL infusate. Given that convection-enhanced delivery generally infuses an area uniformly, and passive application as we did here mainly exposes the surface of the tissue slice to virus, we think that ~10-20% of our tissue slice is seeing the virus at its highest concentration, and so a fraction of the 10-20 mg slice (around 1-3 mg) is truly being dosed by the 2.1E9, similar to the median vg/mg in convection-enhanced trials.

Supplemental Table 2

Trial	NCT	Low estimate, vector genomes per mg brain infused*	High vector genomes per mg brain infused**
NTN Phase 1a, 2a	NCT00252850	1.05E+09	3.16E+09
NTN Phase 1b, 2b	NCT00252850	9.71E+08	2.91E+09
VY-AADC01 phase 1 UCSF cohort 1	NCT01973543	5.39E+08	1.62E+09
VY-AADC01 phase 1 UCSF cohort 2	NCT01973543	5.39E+08	1.62E+09
VY-AADC01 phase 1 UCSF cohort 3	NCT01973543	1.69E+09	5.07E+09
GAD phase 1 2007 Lancet low dose	NCT00195143	4.47E+08	1.34E+09
GAD phase 1 2007 Lancet mid dose	NCT00195143	1.34E+09	4.02E+09
GAD phase 1 2007 Lancet high dose	NCT00195143	4.47E+09	1.34E+10
GAD phase 2	NCT00643890	3.24E+07	9.71E+07
Tay Sachs Phase 1/2 abstract CNS 2023	NCT04669535	1.06E+10	3.17E+10
NTN Sangamo, AAV2-NRTN in PD	NCT00985517	2.16E+09	6.47E+09
MPS, rh.10, SGSH	NCT03612869	3.24E+08	9.71E+08
**Median**		**1.01E+09**	**3.03E+09**
Mean		2.01E+09	6.03E+09

*Low estimate of vg/mg tissue: (#vector genomes infused)/(uL of infusate * 3 * 1.03mg)
**High estimate of vg/mg tissue: (#vector genomes infused)/(uL of infusate * 1 * 1.03mg)
